# Sterigmatocystin-Induced DNA Damage Triggers G_2_ Arrest via an ATM/p53-Related Pathway in Human Gastric Epithelium GES-1 Cells In Vitro

**DOI:** 10.1371/journal.pone.0065044

**Published:** 2013-05-21

**Authors:** Donghui Zhang, Yu Cui, Haitao Shen, Lingxiao Xing, Jinfeng Cui, Juan Wang, Xianghong Zhang

**Affiliations:** 1 Laboratory of Pathology, Hebei Medical University, Shijiazhuang, China; 2 Department of Pathology, The Second Hospital, Hebei Medical University, Shijiazhuang, China; German Cancer Research Center, Germany

## Abstract

Sterigmatocystin (ST), which is commonly detected in food and feed commodities, is a mutagenic and carcinogenic mycotoxin that has been recognized as a possible human carcinogen. Our previous study showed that ST can induce G_2_ phase arrest in GES-1 cells *in vitro* and that the MAPK and PI3K signaling pathways are involved in the ST-induced G_2_ arrest. It is now widely accepted that DNA damage plays a critical role in the regulation of cell cycle arrest and apoptosis. In response to DNA damage, a complex signaling network is activated in eukaryotic cells to trigger cell cycle arrest and facilitate DNA repair. To further explore the molecular mechanism through which ST induces G_2_ arrest, the current study was designed to precisely dissect the role of DNA damage and the DNA damage sensor ataxia telangiectasia-mutated (ATM)/p53-dependent pathway in the ST-induced G_2_ arrest in GES-1 cells. Using the comet assay, we determined that ST induces DNA damage, as evidenced by the formation of DNA comet tails, in GES-1 cells. We also found that ST induces the activation of ATM and its downstream molecules, Chk2 and p53, in GES-1 cells. The ATM pharmacological inhibitor caffeine was found to effectively inhibit the activation of the ATM-dependent pathways and to rescue the ST-induced G_2_ arrest in GES-1 cells, which indicating its ATM-dependent characteristic. Moreover, the silencing of the p53 expression with siRNA effectively attenuated the ST-induced G_2_ arrest in GES-1 cells. We also found that ST induces apoptosis in GES-1 cells. Thus, our results show that the ST-induced DNA damage activates the ATM/53-dependent signaling pathway, which contributes to the induction of G_2_ arrest in GES-1 cells.

## Introduction

It has been shown that sterigmatocystin (ST), which is mainly produced by several Aspergillus species, such as A. *versicolor*, A. *chevalieri*, A. *ruber*, A. *amstelodami*, and A. *aureolatus*, is a quite frequent contaminant in grains, corn, bread, cheese, spices, animal feed, and damp indoor environments [1], [2]. Being structurally related to aflatoxins, ST exposure could induce tumors in several organs in different animals [3]–[5], and ST has been recognized as a possible human carcinogen by the International Agency for Research on Cancer [6]. Several recent *in vivo* studies have shown that the long-term administration of sterigmatocystin can induce intestinal metaplasia in the gastric mucosa of Mongolian gerbils [7], [8]. Our previous study showed that ST can induce G_2_ arrest in human gastric epithelial GES-1 cells *in vitro* and that the JNK, ERK, and PI3K/AKT/mTOR pathways participate in the G_2_ arrest [9].

The cell cycle G_2_ phase arrest is frequently the result of a DNA damage interaction. Because all organisms are continually exposed to environmental and metabolic factors that cause DNA damage, eukaryotic cells have developed elaborate cell cycle checkpoint controls and DNA repair mechanisms to arrest the cell cycle until the damage is repaired [10], [11]. However, if cells cannot repair the damage during cell cycle arrest, the perturbations of cell cycle progression by DNA damage often result in cell death or apoptosis during or after the G_2_ arrest [12]. The activation of cell cycle checkpoints in response to various types of DNA damage is essential for the maintenance of genomic stability in eukaryotic cells [13]. Mutations and/or acquired defects induced by DNA damage are thought to underlie the development and progression of cancer [14], [15]. It has become clear that the response to DNA damage is a signal transduction pathway that involves sensors for lesions, transducer molecules, and a variety of effector molecules. As a member of the phosphoinositide 3-kinase (PI3K) cell signaling family, the Ataxia Telangiectasia Mutated (ATM) kinase is an important sensor activated in the response to DNA damage. ATM, which is triggered by double-strand breaks in DNA (DSBs), initiates a signaling cascade to regulate the cell cycle. Once activated, ATM phosphorylates various downstream molecules including the checkpoint kinase Chk2 and the tumor suppressor protein p53 [16], [17]. Despite our previous study showed that ST-induced PI3K signaling pathway participates in the G_2_ cell cycle arrest in GES-1 cells, the importance of DNA damage and the ATM-dependent pathway in the ST-induced G_2_ phase arrest in GES-1 cells is not yet elucidated .

The p53 transcription factor, which is an important molecule downstream of ATM, plays a key role in the modulation of gene expression programs and cell cycle arrest [18], [19]. Several studies have shown that p53 plays important roles in the regulation of the DNA damage-induced cell cycle arrest [20]–[22]. Nam *et al.* found that the activation of ATM/p53-dependent DNA damage pathway is involved in the etoposide-induced G_2_/M arrest in neural progenitor cells *in vivo* [23]. Xie *et al.* reported that ST can induce G_2_/M phase arrest in murine fibroblasts via the loss of p53-mediated G_1_ checkpoint [24]. Thus, it is necessary to investigate the exact effects of the ATM-downstream molecule p53 on the ST-induced G_2_ arrest in GES-1 cells.

In the present study, we evaluated the effects of ST on DNA damage and the activation of ATM pathway in human gastric epithelium GES-1 cells *in vitro*. We also evaluated the role of ATM/p53-related signaling in the regulation of the ST-induced G_2_ arrest using the ATM inhibitor caffeine and transfecting p53 siRNA into GES-1 cells. In addition, we measured the resultant apoptosis in ST-treated GES-1 cells. Based on our previous findings, this study will provide new insights into the molecular mechanism of ST-induced G_2_ phase arrest in GES-1 cells.

## Materials and Methods

### Chemicals and reagents

Highly purified ST (>99% purity, benzene-free), which was purchased from Sigma-Aldrich (S3255, St. Louis, MO, USA), was dissolved in dimethylsulfoxide (DMSO) to a concentration of 5 mM as the primary stock solution and stored at 4°C. Caffeine was purchased from Alfa Aesar (MA, USA) and dissolved in sterilized water to a concentration of 100 mM as the primary stock solution and stored at −20°C. The primary antibodies used for the Western blot analysis were mouse anti-human Cyclin B1 antibody (eBioscience, CA, USA), rabbit anti-human Cdc2, Cdc25C, ATM, phospho-ATM (Ser-1981), and phospho-Chk2 (Thr-68) monoclonal antibodies (Epitomics, CA, USA), rabbit anti-human Chk2 monoclonal antibodies (Millipore, MA, USA), rabbit anti-human phospho-Cdc2 (Tyr15) and phospho-Cdc25C (Ser216) monoclonal antibodies (Cell Signaling Technology, MA, USA), mouse anti-human phospho-p53 (Ser15) monoclonal antibody (Cell Signaling Technology, MA, USA), rabbit anti-human p53, Bax, and caspase-3 antibodies, and mouse anti-human p21^ waf1^ and Bcl-2 antibodies (Santa Cruz, CA, USA).

### Cell culture and treatment

GES-1 cells derived from a human fetal gastric mucosa epithelium, were purchased from the Beijing Institute for Cancer Research. The cells were cultured in Dulbecco's Modified Eagle Medium (DMEM, Gibco, Carlsbad, California, USA) supplemented with 10% FBS, penicillin (100 U/ml), and streptomycin (100 µg/ml) and maintained at 37°C with 5% CO_2_. All of the experiments were performed on logarithmically growing cells. The cells were treated with solvent (DMSO, at a final solvent concentration of 0.06%(v/v)) alone or with 0.075, 0.3, 1.5 or 3 µM ST for 48 h. In addition, 0.06% (v/v) DMSO was used as the solvent control.

### MTT assay

The MTT method employed to evaluate the level of proliferation. The cells were seeded on 96-well culture plates at a density of 4×10^3^ cells/well and treated with ST at concentrations ranging from 0.03 to 48 µM for 24 h, 48 h, and 72 h at 37°C. At the end of the treatment, 20 µl of the MTT stock solution was added to each well (to obtain a final concentration of 0.5 mg/ml), and the plates were incubated for an additional 4 h. The medium was then replaced with 150 µl of DMSO to dissolve the converted purple dye in the culture plates. The absorbance was measured on a spectrophotometer microplate reader at a wavelength of 560 nm. After correcting for the background absorbance, the cell viability was assayed as the relative formation of formazan in the treated wells compared with the control wells [(A_560_ of treated wells/A_560_ of control wells)×100%].

### Comet assay

The alkaline comet assay, which is a single cell gel electrophoresis assay, is used to sensitively detect single and/or double-strand breaks in DNA, as shown by Singh *et al.* [25], [26]. Briefly, GES-1 cells were exposed to DMSO (0.06%) or 0.075, 0.3, 1.5 or 3 µM ST in DMSO for 48 h. After treatment, the cells were harvested, mixed with 0.75% low-melting point agarose (Bio Basic Inc., NY, USA), and layered onto microscope slides precoated with normal-melting point agarose (0.5% w/v in PBS). The cells were then dissolved in freshly prepared ice-cold lysis buffer (10% DMSO and 1% Triton-X in an alkaline lysis solution composed of 2.5 M NaCl, 10 mM Tris, and 100 mM Na_2_EDTA, pH 10) for 2 h at 4°C in the dark. The slides were placed in a horizontal gel electrophoresis chamber and incubated in alkaline buffer solution (300 mM NaOH and 1 mM Na_2_EDTA, pH>13) for 20 min at 4°C to facilitate DNA unwinding. Electrophoresis was performed in the same buffer at 4°C for 20 min at 20 V and approximately 160 mA. The slides were then washed twice in neutralizing buffer (0.4 M Tris-HCl, pH 7.4) for 5 min. The DNA was stained through the addition of 25 µl of ethidium bromide (20 µg/ml) to each slide.

The comets were visualized at 200× magnification with a fluorescence microscope (Olympus, Tokyo, Japan) equipped with a 530-nm excitation filter. To quantify the induced DNA damage, 200 randomly selected cells (70 cells from each of three replicate slides) from each sample were analyzed with the Comet Assay Software Project (CaspLab-Comet Assay Software, CASP) 1.2.2 (University of Wroclaw, Poland). The DNA damage parameters, including the percent of DNA in the tail (%Tail DNA), the tail length and the Olive tail moment, were calculated from at least 100 cells. All of the slides were coded to prevent observer bias. The non-parametric Mann-Whitney U test was used to compare the DNA damage between the solvent-treated control cells and the ST-treated cells.

### Cell cycle analysis

The cells were cultured as described above and harvested in buffer (0.05% trypsin in PBS). After centrifugation for 5 min at 1000 rpm and 4°C, the cells were resuspended in cold 70% ethanol and stored at 4°C overnight. After resuspension, the cells were washed and incubated with RNase A (2 mg/ml) at 37°C for 30 min, and stained with 50 µg/ml propidium iodide (PI, Sigma, USA) containing 0.1% Triton X-100 and 0.02 mg/ml EDTA at 37°C for 15 min. The flow cytometry (FCM) analysis was performed using a FACS Calibur (Becton Dickinson, USA).

### Annexin V/PI flow cytometric staining assay

The apoptosis of GES-1 cells was detected using the Annexin V-FITC kit (MultiSciences Biotech., Hangzhou, China) according to the manufacturer's protocol. The cells were cultured as described above, treated with different concentration of ST (0.075, 0.3, 1.5, and 3 µM) for 48 h, collected, and then washed with ice-cold buffer (0.05% trypsin in PBS). To detect early and late apoptosis, both adherent and suspension cells were harvested together. The washed cell pellet was resuspended in ice-cold 1× binding buffer containing FITC-conjugated Annexin V and PI. The sample was incubated for 5 min in the dark before it was analyzed using a flow cytometer (Epics-XLII). This assay discriminates between intact cells (Annexin V−/PI−), early apoptotic cells (Annexin V+/PI−), and late apoptotic/necrotic cells (Annexin V+/PI+).

### Morphological analysis of apoptotic cells

The morphological changes in the nuclear chromatin of cells undergoing apoptosis were detected by staining with 2 µg/ml Hoechst 33258 fluorochrome (Molecular Probe). The stained cells were then examined under a fluorescence microscope (Olympus, Japan).

### siRNA transfection

A siRNA against human p53 was synthesized by GenePharma Co., Ltd. (Shanghai, China). The siRNA sequences targeting p53 were 5′-CUA CUU CCU GAA AAC AAC GdT dT-3′ and 5′-CGU UGU UUU CAG GAA GUA GdT dT-3′. The GES-1 cells were transfected with siRNA at a final concentration of 60 nM in 6-well plates using Lipofectamine 2000 according to manufacturer instructions (Invitrogen, Carlsbad, CA, USA). Twenty-four hours post transfection,the efficiency of inhibition at the p53 mRNA level was estimated to be approximately 85% by real-time PCR. The medium was also discarded 24 h post transfection, and the cells were washed with PBS and subsequently treated with 3 µM ST for 48 h. The cells were then harvested and assayed by Western blot and flow cytometry.

### Western blot analysis

The whole cell protein from GES-1 cells was extracted using lysis buffer (1% Triton X-100, 150 mM NaCl, 2 mM EDTA, 50 mM Tris-HCl, and 1% cocktail). The protein (60 µg/lane) was used for SDS-PAGE and transferred to a PVDF membrane after electroblotting at 4°C. Subsequently, the membranes were blocked with 5% nonfat milk at 37°C, incubated with primary antibody at 4°C overnight, and then incubated with peroxidase conjugated secondary antibody for 1.5 h at 37°C. The protein bands were visualized using an enhanced chemiluminescence (ECL) system, quantified by densitometry using Syngene Image Systems and normalized to β-actin.

### Statistical analysis

All of the data and results were confirmed through at least three independent experiments. The values shown represent the means ± standard deviation (SD). The significance of the differences was identified through one-way analysis of variance (ANOVA). The dose-effect relationship was analyzed through correlation and regression analysis. All of the statistical analyses were performed using the SPSS 13.0 statistical software. Differences with a p value less than 0.05 were considered significant.

## Results

### Time- and dose-dependent inhibition of the growth of ST-treated GES-1 cells

**Figure 1:**
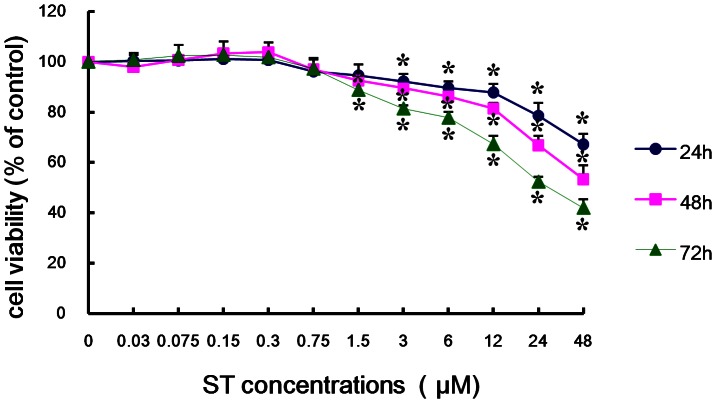
Dose- and time- dependent effects of ST on the viability of GES-1 cells. The cell viability was determined by the MTT assay after the cells were exposed to ST concentrations in the range of 0.03 to 48 µM for 24, 48, and 72 h. The data represent the means ± SD of three independent experiments. *P<0.05 compared with the control group.

To evaluate the cytotoxic effects of ST on GES-1 cells, we treated cells with ST at concentrations ranging from 0.03 to 48 µM for 24, 48, and 72 h (Fig. 1). We demonstrated that an ST dose in the range of 1.5 µM to 48 µM was cytotoxic for cells even when the cells were only treated for 48 h and that this cytotoxicity is dose-dependent (r = −0.955, *P*<0.01). A similar result was obtained after 72 h of exposure to ST, although higher percentages of cell death were observed (r = −0.913, *P*<0.01). A longer exposure to 24 µM and 48 µM ST significantly decreased the cell viability, and this decrease was found to be time-dependent (r_24 µM_ = −0.998, r_48 µM_ = −0.998, *P*<0.05). Therefore, we concluded that ST inhibits cellular proliferation in a dose- and time-dependent manner. Based on these preliminary results, we choose the time point of 48 hours and the ST treatment concentration range of 0.075 to 3 µM for the subsequent experiments.

### ST induced DNA damage in GES-1 cells

**Figure 2:**
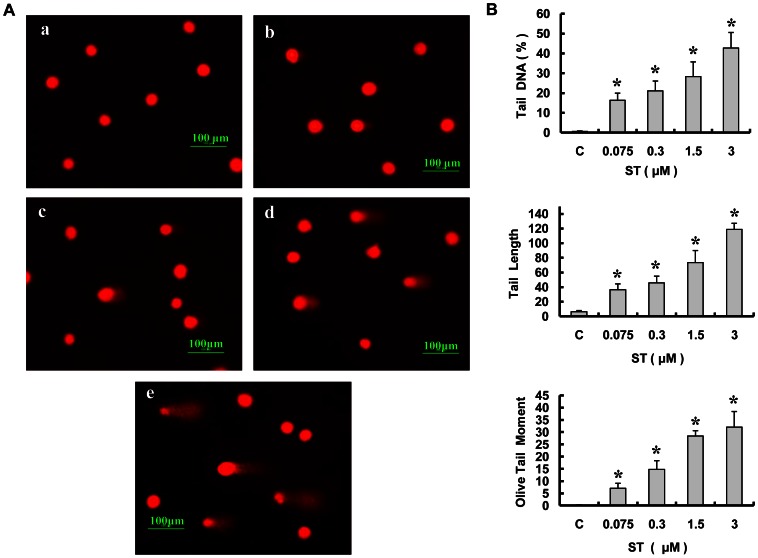
ST induces DNA damage in GES-1 cells. Cells were treated with 0.06% DMSO or different concentrations of ST (0.075, 0.3, 1.5, and 3 µM) in DMSO and then subjected to the comet assay as described in Section 2. (A) Cells containing DNA strand breakage (with long tails) were observed under an inverted fluorescence microscope and quantified. (200× magnification; *n* = 3). The data shown are representative of at least three separate experiments. (B) The ST-induced DNA damage was characterized by an increase in the percentage of DNA tail, the Ttail length, and the Olive tail moment in GES-1 cells. The following groups were assayed: (a) solvent control, (b) 0.075 µM ST, (c) 0.3 µM ST, (d) 1.5 µM ST, and (e) 3 µM ST. The data represent the means ± SD. Differences were considered statistically significant if *P<0.01 according to the non-parametric Mann-Whitney U test.

The comet assay was performed under alkaline conditions for the detection of a broad spectrum of DNA lesions, including DNA double-strand breaks (DSBs), DNA single-strand breaks, and alkaline-labile sites [27], [28]. We examined the effect of ST treatment on DNA damage in GES-1 cells using the alkaline comet assay in individual cells. Almost all of comets in the control cells showed no fluorescent tails, which indicates that the nuclear DNA was intact. In contrast, the exposure of the cells to different concentrations of ST for 48 h increased the number of typical comets with tails of different fluorescence intensities, which is an evident indicator of DNA strand breakage (Fig. 2A). The values of the %Tail DNA, the tail length, and the Olive tail moment were significantly increased in the ST-treated groups compared with the solvent-treated control group (Fig. 2B). In addition, these increases were found to be dose-dependent (r = 0.952, 0.965, and 0.938 *P*<0.05). These results suggest that ST can induce DNA damage in GES-1 cells.

### Activation of the ATM-Chk2 pathway in ST-treated GES-1 cells

**Figure 3:**
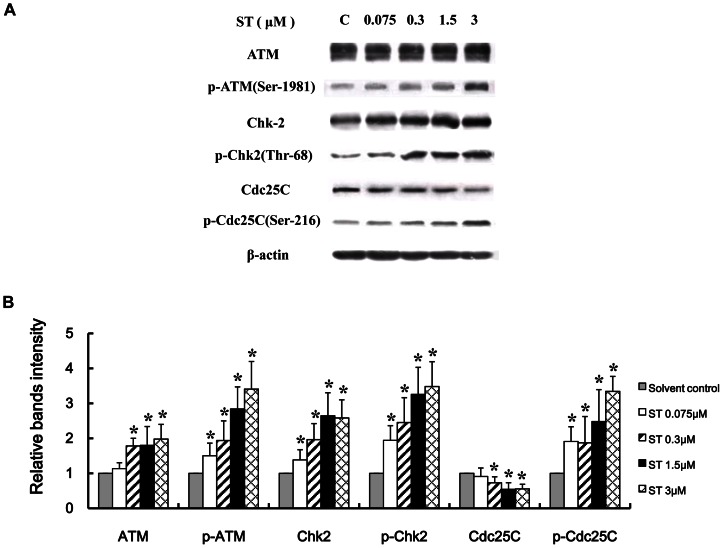
ATM-Chk2 signaling pathway is activated in ST-treated GES-1 cells. GES-1 cells were treated with different concentrations of ST (0.075, 0.3, 1.5, and 3 µM) or solvent for 48 h. (A) Representative immunoblots show the effect of ST treatment on the phosphorylation of ATM (Ser-1981), Chk2 (Thr-68), and Cdc25C (Ser-216) and the expression of ATM, Chk2, and Cdc25C. β-actin was used as the normalization control. (B) Intensities of the immunoreactive bands were quantified by densitometric scanning and compared with those of the control (considered “1”). The values shown represent the means ± SD. *P<0.05 compared with the solvent-treated control group.

We investigated the activation of ATM in ST-treated GES-1 cells by Western blot. As shown in Fig. 3, the phosphorylation level of ATM (Ser-1981) and the total ATM expression in GES-1 cells treated with 0.3 to 3 µM ST were significantly higher compared with those in the solvent-treated control group (*P*<0.05). These results indicate that the ST-induced DNA damage might activate the ATM signaling pathway in GES-1 cells. We then found that ST activates Chk2, which is an effector downstreamof ATM; this finding was evidenced by the increased phosphorylation level of Chk2 (Thr-68) and the total Chk2 expression in GES-1 cells (Fig. 3). In addition, we measured the expression of the cell cycle regulatory protein Cdc25C, which is a signaling molecule downstream of Chk2. ST induced an increase in the phosphorylation level of Cdc25C (Ser-216) and decreased the total Cdc25C expression in GES-1 cells (Fig. 3). These data indicate that the ST-induced DNA damage might activate the ATM-Chk2 checkpoint signaling pathways in GES-1 cells.

### Activation of the p53-p21 pathway in ST-treated GES-1 cells

**Figure 4:**
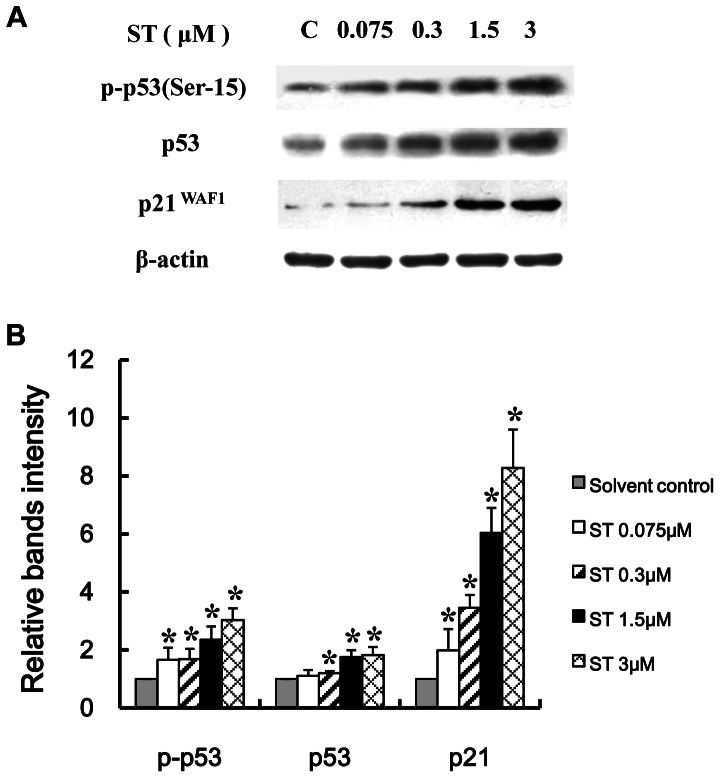
The p53-p21 pathway is activated in ST-treated GES-1 cells. GES-1 cells were treated with different concentrations of ST (0.075, 0.3, 1.5, and 3 µM) or solvent for 48 h. (A) Representative immunoblots show the effect of ST treatment on the phosphorylation of p53 (Ser-15) and the expression of p53 and p21. β-actin was used as the normalization control. (B) Intensities of the immunoreactive bands were quantified by densitometric scanning and compared with those of the control (considered “1”). The values shown represent the means ± SD. *P<0.05 compared with the solvent-treated control group.

The activation of p53 plays critical roles in the cellular responses to DNA damage and the regulation of the cell cycle progression. Furthermore, p53 is a key molecule downstream of the ATM kinase and is considered to be triggered by the activation of ATM. We therefore examined the activation of p53 in GES-1 cells treated with ST for 48 h. The Western blot analysis results showed that ST significantly increased the expression of phosphorylated p53 (Ser-15) and total p53 (*P*<0.05, Fig. 4). We alsofound that ST increased the expression of the p53 transcriptional target p21^waf1^ in GES-1 cells (*P*<0.05, Fig. 4). These data suggest that the ST-induced DNA damage might activate the signaling pathway downstream of p53-p21 in GES-1 cells.

### ATM-dependent pathways regulating ST-induced G_2_ arrest in GES-1 cells

**Figure 5:**
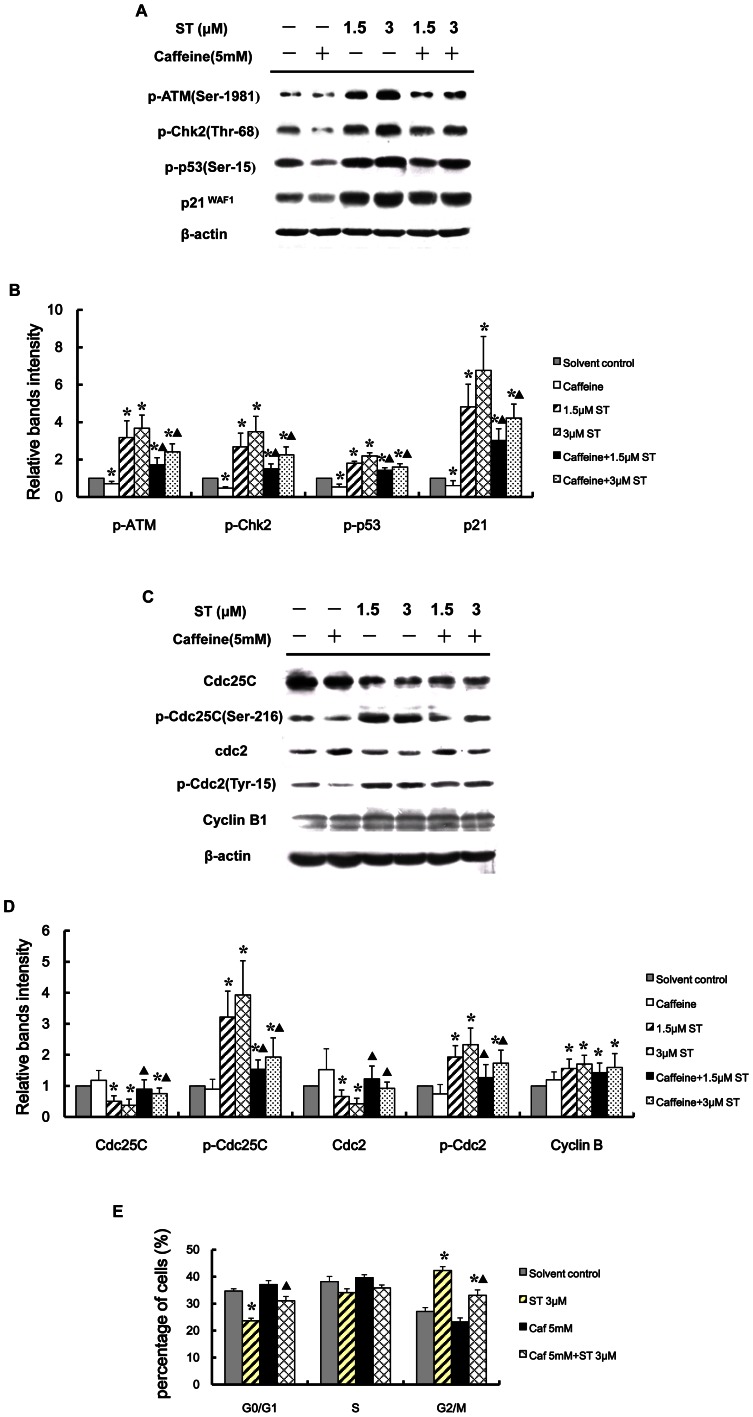
ATM inhibitor (caffeine) attenuates ST-induced G_2_ arrest in GES-1 cells. Cells were treated with the indicated agent for 48 h (pretreatment with 5mM caffeine for 2 hours followed by ST treatment). (A) Caffeine blocked the phosphorylation of ATM (Ser-1981), Chk2 (Thr-68), and p53 (Ser-15) and downregulated the expression of p21 stimulated by ST exposure. (C) Caffeine affected the G_2_/M phase regulatory proteins that were altered by ST treatment. β-actin was used as the loading control. (B, D) Intensities of the immunoreactive bands in “A” and “C” were quantified by densitometric scanning and compared to those of the control (considered “1”). (E) Caffeine effectively prevented the G_2_ arrest induced by ST, as demonstrated by flow cytometric analysis. The data represent the means ± SD of three independent determinations. *P<0.05, compared with the solvent-treated control group. **^▴^** *P*<0.05 compared with the ST-treated group.

To further confirm that the activation of the ATM-dependent pathway contributes to the ST-induced G_2_ arrest, the well-known ATM inhibitor caffeine was used in this study. Western blotting revealed that the ST-induced ATM activation was effectively inhibited by 5 mM caffeine (Figs. 5A and 5C). In addition, caffeine also significantly decreased the phosphorylation levels of Chk2 (Thr68), p53 (Ser15), and p21 in ST-treated GES-1 cells (*P*<0.05, Figs. 5A and 5C). The results indicate that the ST-induced DNA damage activates the ATM-Chk2 and ATM-p53 signaling pathways in GES-1 cells.

More importantly, as shown in Fig. 5E, the flow cytometric analysis results showed that the proportion of cells in G_2_/M phase in the caffeine pretreatment group was 33.10±1.99%, which is significantly lower than that in observed in the group treated with ST alone (42.30±1.42%, *P*<0.05). This result suggests that the activation of ATM signaling pathway contributes to the ST-induced G_2_ arrest. Furthermore, we found that caffeine prevented the ST-induced alterations in the expression of Cdc25C and Cdc2 in GES-1 cells (Figs. 5B and 5D). However, the pretreatment with caffeine did not affect the ST-increased protein level of Cyclin B1. Collectively, these results suggest that the signaling pathways downstream of ATM play a predominant role in the regulation of the ST-induced G_2_ arrest.

### p53 is required for ST-induced G_2_ arrest in GES-1 cells

**Figure 6:**
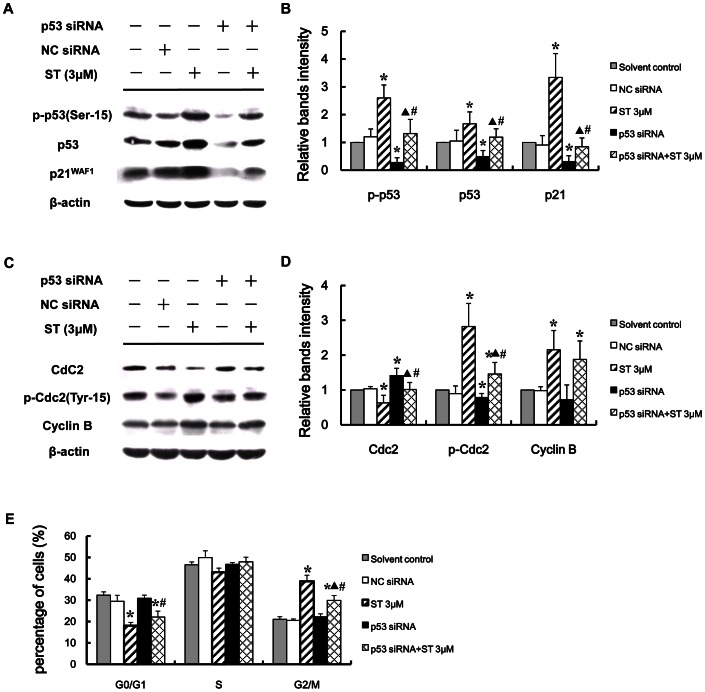
Silencing of p53 by specific p53 siRNA inhibited ST-induced G_2_ arrest. Cells were either not transfected or transfected with 100 nM p53 siRNA and then treated with 3 µM ST for 48 h. (A) Cells were subjected to immunoblot analysis for p-p53 (Ser15), p53, p21, and (C) the regulators related to G_2_ arrest. NC: cells transfected with the same concentration of negative control siRNA. β-actin was used as the loading control. (B, D) Intensities of the immunoreactive bands in “A” and “C” were quantified by densitometric scanning and compared with those of the control (considered “1”). (E) The cell cycle phases of the cells were analyzed by FCM. The values shown represent the means ± SD, *P<0.05 compared with the solvent-treated control group. **^▴^** *P*<0.05 compared with the ST-treated groups. ^#^ *P*<0.05 compared with the p53 siRNA-treated groups.

To further explore the role of p53 in the ST-induced G_2_ arrest, we knocked down p53 in GES-1 cells using its respective specific siRNA and then subjected the cells to ST (3 µM) treatment for 48 h. The Western blotting results showed that the p53-targeting siRNA effectively downregulated the expression of phosphorylated p53, total p53, and p21^waf1^ in the ST-treated GES-1 cells (Figs. 6A and 6C). In addition, the FCM analysis showed that the blocking of p53 signaling by siRNA transfection decreased the G_2_ population from 38.83% to 29.90% in response to treatment with ST (Fig. 6E). These results indicate that the blocking of p53 inhibits the ST-induced G_2_ arrest in GES-1 cells. We also observed the effects of ST on the expression of the G_2_/M phase regulatory proteins in GES-1 cells transfected with the p53 siRNA. As shown in Figs. 6B and 6D, the transfection of the p53 siRNA upregulated Cdc2, downregulated phosphorylated Cdc2, and did not affect the level of Cyclin B1 in ST-treated GES-1 cells. Taken together, these data indicate that p53 participates in the regulation of the ST-mediated G_2_ phase arrest in GES-1 cells through the downregulation of the expression of p21.

### ST induced apoptosis in GES-1 cells

**Figure 7:**
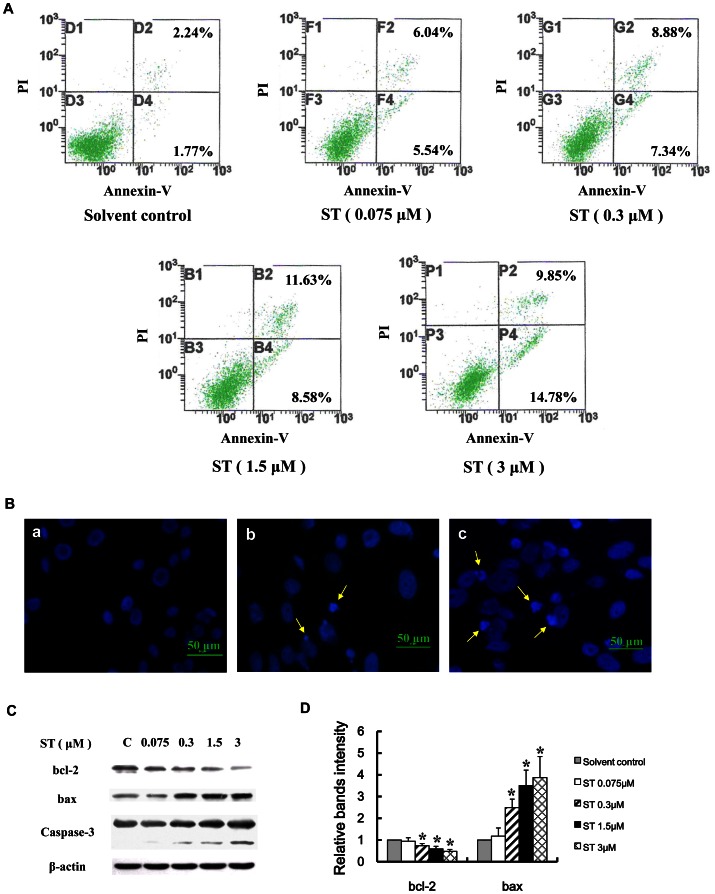
ST induces apoptosis in GES-1 cells. GES-1 cells were treated with the indicated agents for 48 h. (A) Flow cytometric analysis of ST-induced apoptosis using Annexin V-FITC/PI. The living, early apoptotic, late apoptotic/necrotic, and damaged cells are present in the lower left, lower right, upper right, and upper left quadrants, respectively. (B) Fluorescent staining of nuclei in ST-treated and untreated cells by Hoechst 33258. Cells were visualized with a fluorescence microscope. The following groups were assayed: (a) solvent control, (b) 1.5 µM ST, and (c) 3 µM ST. Condensed and fragmented nuclei and apoptotic bodies were observed in the ST-treated cells, but not in the solvent-treated control cells. (C) Western blot analysis of the effect of the ST dosage on mitochondria-dependent apoptosis-related proteins. Representative immunoblots show the effect of ST on the expression of Bcl-2 and Bax and the activation of caspase-3. β-actin was used as the normalization control. (D) Intensities of the immunoreactive bands in “C” were quantified by densitometric scanning and compared with those of the control (considered “1”). The values shown represent the means ± SD, *P<0.05 compared with the solvent-treated control group.

To assess whether apoptosis contributes to the growth inhibition observed in ST-treated cells, we evaluated the effects of ST on apoptosis in GES-1 cells. As shown in Fig. 7A, the percentage of apoptotic cells was increased significantly in the ST-treated groups (0.075, 0.3, 1.5 and 3 µM) compared with that in the solvent-treated control group. Moreover, the levels of chromatin condensation and fragmentation, which are typical nuclear morphological changes that are found in apoptotic cells, were monitored by Hoechst 33258 staining in GES-1 cells treated with 1.5 and 3 µM ST for 48 h (Fig. 7B).

To further characterize the apoptotic pathway activated by ST, we investigated the effects of ST on the caspase activity and the expression of proteins that are pivotal for apoptosis, including Bcl-2 and Bax. As shown in Fig. 7C, ST dose-dependently inducted of a cleaved form of caspase-3. In addition, the upregulation of Bax and the downregulation of Bcl-2 were observed, which suggests that an increase in the Bax/Bcl-2 ratios might be involved in the apoptosis pathways induced by ST in GES-1 cells. The above findings confirm that ST induces apoptosis in GES-1 cells.

## Discussion

It is generally accepted that the induction of cell-cycle arrest is an important biological effect of many carcinogenic mycotoxins [29], [30]. Several mycotoxins have been identified to induce G_2_/M phase arrest [31], [32]. Our recent report showed that ST treatment can induce cell cycle arrest at the G_2_ phase in GES-1 cells *in vitro* and thatthe activation of the MAPK and PI3K signaling pathways is involved in the G_2_ phase arrest [9]. To further explore the possible molecular mechanisms in ST-induced G_2_ phase arrest, we evaluated the effects of DNA damage and the ATM signaling cascade on the ST-induced G_2_ arrest in GES-1 cells. The results showed that ST can induce DNA damage and subsequently activate ATM-Chk2 and ATM-p53 signaling pathways. The blocking of the ATM pathway effectively attenuated the ST-induced G_2_ arrest in GES-1 cells. We also found the inhibition of p53 expression could prevent the ST-induced G_2_ arrest. These results clearly demonstrate that the ST-induced DNA damage triggers G_2_ arrest through the ATM/p53-dependent signaling pathways in GES-1 cells. Thus, the mechanism by which the ST-induced DNA damage results in G_2_ arrest is similar to those induced by ionizing radiation and chemicals such as naphthalimides, kotomolide A *et al.* [33]–[35]. In addition, the results show that ST can induce apoptosis in GES-1 cells.

In the presence of DNA damage, a number of checkpoint pathways are activated to arrest the cells at G_1_/S, S, or G_2_/M transitions. This arrest provides time for DNA repair, which results in the minimization of the replication and/or induction of the segregation of damaged DNA or apoptosis if the cellular damage cannot be properly repaired [36], [37]. Unrepaired or inappropriately repaired DNA damage can lead to mutagenic events, such as chromosome loss, deletions, duplications, and translocations. The disruption of normal checkpoint function from inherited and acquired genetic mutations is increasingly recognized as a pathophysiological mechanism responsible for tumor-prone human disease syndromes [14], [38], [39]. The G_2_/M checkpoint is often activated by DNA damage lesions, especially DNA double-strand breaks (DSBs). A number of independent studies have reported that ST can cause DNA damage and form DNA adducts, which lead to chromosome aberration and sister-chromatid exchange in animal experiments [40], [41], [42]. Moreover, our previous study speculated that a possible mechanism through which ST can induce the activation of the ERK, JNK, and PI3K/AKT/mTOR pathways might depend on DNA damage [9]. However, there was no direct evidence that ST induced DNA damage in GES-1 cells. In this work, we found that ST significantly induced DNA strand breakage in human gastric epithelial GES-1 cells in a dose-dependent manner through the generation of “comet tails”. This finding indicates that the G_2_ cell cycle arrest induced by ST might occur in a population of damaged GES-1 cells that can potentially undergo cell death or apoptosis unless this DNA damage is partially or completely fixed.

Several highly conserved proteins are recruited to damaged DNA for checkpoint activation. In general, DNA damage-induced signaling is initiated by the DNA damage sensor ATM kinase, which is a member of the PI3K signaling family [43], [44]. It is known the signaling downstream of ATM is commonly triggered by DNA DSBs [45], [46]. In the presence of DSBs, ATM becomes activated and phosphorylates numerous downstream targets, such as Chk2, p53, MDM2, and H2AX, which act as signal transducers and effectors that initiate cell cycle arrest and apoptosis [47], [48]. Recently, Zhu found that the chemicals amonafide and R16 can induce DNA DSBs, which trigger the ATM-activated Chk2-executed pathway and ultimately lead to G_2_ phase arrest, in HCT116 cells [33]. In our study, we showed that ST induces the activation of ATM through its phosphorylation at Ser1981 and subsequently initiates a series of signaling cascades through the activation of Chk2 and p53, which are molecules downstream of ATM. The blocking of the ATM signaling pathway by the inhibitor caffeine prevented the phosphorylation of Chk2 and p53 and attenuated the ST-induced G_2_ arrest in GES-1 cells treated with ST. These findings indicate that ATM and its downstream molecules (Chk2 and p53) likely contribute to the ST-induced G_2_ arrest in GES-1 cells. However, we also found that ATM inhibition does not completely abrogate the ST-induced G_2_ arrest, which suggests that other signaling pathways are also involved in the ST-induced G_2_ arrest in GES-1 cells, as suggested in our previous study [9].

In response to DNA damage, the Thr-68 residue in the N-terminal S/TQ cluster domain of Chk2 is phosphorylated by its upstream kinase ATM [49]. Once activated, Chk2 acts as a diffusible signal transducer and phosphorylates a multitude of substrates involved in cell cycle control, transcription, and apoptosis. Activated Chk2 phosphorylates the Ser-216 residue of Cdc25C, which is an activating phosphatase for Cdc2 that dephosphorylates the Tyr-15 residue of Cdc2 to create a binding site for a 14-3-3 protein that prevents Cdc25C from activating the Cyclin B1/Cdc2 complex and ultimately induces G_2_ phase arrest. Moreover, Chk2 plays an essential role in the maintenance rather than the initiation of G_2_ arrest because Chk2-null ES cells can initiate arrest but cannot maintain long-term arrest [50]. In this study, we found that ST increased the phosphorylation of Chk2 associated with the activation of ATM in response to DNA damage in GES-1 cells. The ATM inhibitor caffeine inhibited the ST-induced activation of Chk2, increased the expression of Cdc25C and Cdc2 and decreased the expression of phospho-Cdc25C and phospho-Cdc2 in ST-treated GES-1 cells. Taken together, these data suggest that caffeine may inhibit the G_2_/M checkpoint by inactivating the ATM-Chk2 pathway. Therefore, it can be concluded that this pathway contributes to the ST-induced G_2_ arrest in GES-1 cells and that the activation of the ATM-Chk2 pathway is involved in the ST-induced G_2_ arrest in GES-1 cells.

The tumor suppressor gene p53 is a key element in the induction of cell cycle arrest and apoptosis in response to DNA damage or cellular stress in human cells [21], [51]. Cell cycle arrest that is dependent on p53 requires the transactivation of p21^waf1/cip1^, which is a cyclin-dependent kinase inhibitor (CdkI) that acts as an inhibitor of cell cycle progression via its ability to inhibit Cdc2 [52]. The induction of p21^waf1/cip1^ results in arrest in the G_2_/M phase through the binding of the Cyclin B1/Cdc2 complex [53], [54]. In this study, we showed that the treatment of GES-1 cells with ST resulted in the accumulation of p53 and phospho-p53 (Ser15) and increased the expression of p21^waf1^ in a dose-dependent manner. Moreover, we also found that the inhibition of the cell cycle progression by ST was partially overridden by the suppression of normal p53 activity via its specific p53 siRNA, which suggests that p53 activation plays an important role in the ST-mediated G_2_ arrest. In addition, the transfection of GES-1 cells with p53 siRNA reduced the ST-induced expression of p21^waf1^ but not Chk2 (data not shown), which confirms that the p53-p21 pathway downstream of ATM is involved in the ST-induced G_2_ arrest. Our results also show that the p53 siRNA slightly increased the level of Cdc2 and significantly reduced the level of phosphorylated Cdc2 in ST-treated GES-1 cells, which suggests that ST treatment induced G_2_ arrest by inactivating Cdc2 through the p53-p21 downstream pathway. In a previous study, we found that the ST-induced up-regulation of Cyclin B1 did not help stabilize the ST-mediated G_2_ arrest and thus presumed that this upregulation might be associated with the carcinogenesis of ST [9]. In this work, the silencing of p53 did not affect the high expression of Cyclin B1 induced by ST, which indicates that the upregulation of Cyclin B1 might have no relationship with the ST-induced activation of p53. In general, it is clear that DNA damage, which induced the activation of the ATM-p53-p21 pathway, was involved in the ST-induced G_2_ arrest in GES-1 cells.

Accumulating evidence indicates that the early toxic effects of many environmental carcinogens lead to apoptosis [55], [56]. To understand the mechanism underlying the cytotoxicity of ST, we measured cell apoptosis in ST-treated GES-1 cells. We used Hoechst 33258 staining and an Annexin V/PI flow cytometric staining assay and found that ST induced apoptosis in GES-1 cells. The changes in the expression of the key proteins Bcl-2 and Bax and the activation of caspase-3 play an important role in the induction of cell apoptosis [56], [57]. Our results show that ST treatment led to the upregulation of Bax and the downregulation of Bcl-2, which results in an increase in the Bcl-2/Bax ratio, and the activation of caspases-3 through cleavage in GES-1 cells. These results further confirm that ST induces apoptosis in GES-1 cells.

**Figure 8:**
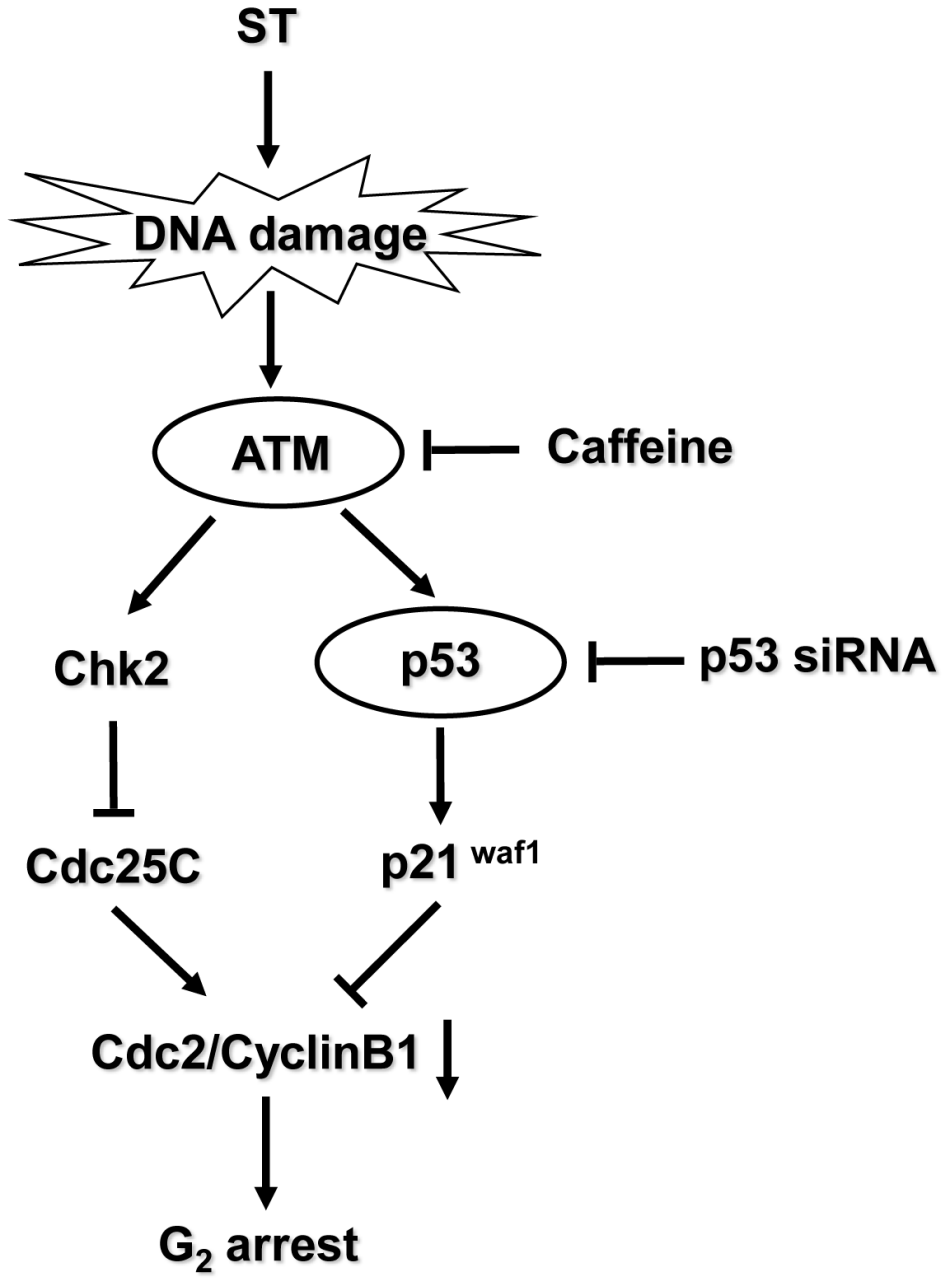
Effect of ST on DNA damage-induced ATM activation and G_2_ arrest in GES-1 cells. In response to ST-induced DNA damage, ATM serves as a signal transducer for the activation of its downstream signaling pathway. Activated ATM simultaneously phosphorylates the Thr-68 and Ser-15 residues of Chk2 and p53, respectively. These phosphorylations lead to the activation of their downstream pathway components, which results in the inhibition of the activation of Cdc25 and an increase in the expression of p21^waf1^. These steps finally result in the inactivation of the Cyclin B1/Cdc2 complex and the induction of G_2_ arrest.

In summary, our present study demonstrates that ST induces DNA damage and subsequently triggers the ATM-activated Chk2- and p53-executed pathways, which contribute to the ST-induced G_2_ phase arrest in GES-1 cells. Thus, besides the involvement of MAPK and PI3K pathways, ATM/p53-related signaling pathway, which is activated by DNA damage, is also involved in the ST-induced G_2_ arrest (Fig. 8). Therefore, our findings provide new insights in the possible carcinogenic mechanism of ST exposure in human gastric epithelial cells.
